# The Association Between Muscle Deoxygenation and Muscle Hypertrophy to Blood Flow Restricted Training Performed at High and Low Loads

**DOI:** 10.3389/fphys.2019.00446

**Published:** 2019-04-17

**Authors:** Thaís M. P. C. Biazon, Carlos Ugrinowitsch, Samuel D. Soligon, Ramon M. Oliveira, João G. Bergamasco, Audrey Borghi-Silva, Cleiton A. Libardi

**Affiliations:** ^1^MUSCULAB – Laboratory of Neuromuscular Adaptations to Resistance Training, Department of Physical Education, Federal University of São Carlos (UFSCar), São Carlos, Brazil; ^2^Escola de Educação Física e Esporte, Universidade de São Paulo (USP), São Paulo, Brazil; ^3^Cardiopulmonary Physiotherapy Laboratory, Physical Therapy Department, Federal University of São Carlos (UFSCar), São Carlos, Brazil

**Keywords:** electromyography, muscle oxygenation, vascular occlusion, muscle hypertrophy, muscle strength

## Abstract

The metabolic stress induced by blood flow restriction (BFR) during resistance training (RT) might maximize muscle growth. However, it is currently unknown whether metabolic stress are associated with muscle hypertrophy after RT protocols with high- or low load. Therefore, the aim of the study was to compare the effect of high load RT (HL-RT), high load BFR (HL-BFR), and low load BFR (LL-BFR) on deoxyhemoglobin concentration [HHb] (proxy marker of metabolic stress), muscle cross-sectional area (CSA), activation, strength, architecture and edema before (T1), after 5 (T2), and 10 weeks (T3) of training with these protocols. Additionally, we analyzed the occurrence of association between muscle deoxygenation and muscle hypertrophy. Thirty young men were selected and each of participants’ legs was allocated to one of the three experimental protocols in a randomized and balanced way according to quartiles of the baseline CSA and leg extension 1-RM values of the dominant leg. The dynamic maximum strength was measured by 1-RM test and vastus lateralis (VL) muscle cross-sectional area CSA echo intensity (CSA_echo_) and pennation angle (PA) were performed through ultrasound images. The measurement of muscle activation by surface electromyography (EMG) and [HHb] through near-infrared spectroscopy (NIRS) of VL were performed during the training session with relative load obtained after the 1-RM, before (T1), after 5 (T2), and 10 weeks (T3) training. The training total volume (TTV) was greater for HL-RT and HL-BFR compared to LL-BFR. There was no difference in 1-RM, CSA, CSA_echo,_ CSA_echo_/CSA, and PA increases between protocols. Regarding the magnitude of the EMG, the HL-RT and HL-BFR groups showed higher values than and LL-BFR. On the other hand, [HHb] was higher for HL-BFR and LL-BFR. In conclusion, our results suggest that the addition of BFR to exercise contributes to neuromuscular adaptations only when RT is performed with low-load. Furthermore, we found a significant association between the changes in [HHb] (i.e., metabolic stress) and increases in muscle CSA from T2 to T3 only for the LL-BFR, when muscle edema was attenuated.

## Introduction

Resistance training- (RT) induced changes in muscle strength are partially due to increases in muscle cross-sectional area (CSA) (i.e., muscle hypertrophy) and changes in muscle architecture (e.g., increase in the pennation angle of muscle fibers) (Aagaard et al., 2001).

It has been widely accepted that RT-induced muscle hypertrophy occurs through two primary mechanisms: mechanical tension and metabolic stress (Schoenfeld, 2010, 2013; Pearson and Hussain, 2015). High-load RT (HL-RT, ∼80% of 1-RM) programs seem to simultaneously activate both mechanisms, increasing muscle protein synthesis and, therefore, muscle hypertrophy (Schoenfeld, 2010). On the other hand, low load blood flow restriction resistance training (LL-BFR) produces similar muscle hypertrophy responses to HL-RT, but metabolic stress seems to be the main mechanism inducing the gains in muscle CSA (Takada et al., 2012; Scott et al., 2014), as mechanical tension is usually low (i.e., 20–50% of the 1 RM load). Accordingly, it has been demonstrated that LL-BFR produces greater changes in blood deoxyhemoglobin concentration [HHb] (i.e., proxy marker of metabolic stress) (Cayot et al., 2016; Lauver et al., 2017), compared to traditional low-load RT (Lauver et al., 2017), suggesting higher metabolic stress in the former than in the last (Cayot et al., 2016; Lauver et al., 2017). The high metabolic stress during LL-BFR (e.g., [P_i_]) is strongly associated with muscle hypertrophy response after only 2 weeks of training (*r* = 0.87) (Takada et al., 2012), indicating that mechanical tension may not be required to induce muscle hypertrophy in high metabolic stress conditions. However, we demonstrated that early increases in muscle CSA (i.e., ∼6–9 RT sessions) are partially due to RT-induced muscle damage edema (Damas et al., 2016b #4). Thus, actual muscle hypertrophy response may take longer to be observed (i.e., ∼20 RT sessions) (Damas et al., 2016a, 2018) weakening Takada et al. (2012) findings, and suggesting that further scrutiny is required.

Our group previously demonstrated that high-load blood flow restriction (HL-BFR) training programs do not produce additive effects compared to traditional HL-RT programs (Laurentino et al., 2008). Thus, it is reasonable to suggest that metabolic stress is required to produce muscle hypertrophy only when mechanical tension is low, as in LL-BFR training programs. Thus, assessing [HHb] during HL-BFR and determining its relationship with muscle hypertrophy response may help shedding light into the role of metabolic stress to muscle adaptive response under high mechanical tension.

Therefore, we compared the effects of HL-RT, HL-BFR, and LL-BFR on muscle [HHb], CSA, activation, strength, architecture, and edema before (T1), after 5 (T2), and 10 weeks (T3) of training. Additionally, we analyzed the association between muscle deoxygenation and muscle hypertrophy. Our hypotheses were (1) BFR would not produce an additive effect on neuromuscular adaptations when mechanical tension is high; (2) high mechanical tension protocols (i.e., HL-RT and HL-BFR) would not present an association between metabolic stress and muscle hypertrophy; (3) low load BFR training would produce similar neuromuscular adaptations to HL-RT; and (4) metabolic stress would have an association with muscle hypertrophy only when mechanical tension is low (i.e., LL-BFR).

## Materials and Methods

### Participants

Thirty young men volunteered to participate in the present study (age: 22 ± 3 years; body mass: 72.7 ± 10.7; kg; height: 178 ± 5 cm; BMI: 22.81 ± 2.99 kg⋅m^2^. Inclusion criteria were: (*i*) Not be engaged in regular resistance training and/or endurance training for at least 6 months prior to commencement of the experimental period; (*ii*) being free of cardiovascular and neuromuscular disorders; (*iii*) having a BMI < 30 kg⋅m^2^; and (*iv*) do not use supplements, anti-inflammatory medications and anabolic steroids.

### Experimental Design

The present randomized controlled trial used a prospective, single-group, intra-subject design in which each leg of the subjects was exposed to one of three experimental protocols. Before the commencement of the experimental protocol, participants engaged into two familiarization sessions to get acquainted with the training protocol and testing procedures. Familiarization sessions were interspaced by 72 h. Seventy-two hours after the last familiarization session, leg extension 1-RM test was performed. Ninety-six hours after the 1-RM test, vastus lateralis (VL) muscle CSA, echo intensity (CSA_echo_), pennation angle (PA) were assessed by ultrasonography (US). Each of the participants’ legs was allocated to one of the three experimental protocols in a randomized and balanced way according to baseline CSA and leg extension 1-RM values of the dominant leg. In short, participants’ legs were divided into quartiles according to muscle CSA and 1-RM values; afterward, legs within each quartile were randomly allocated into the three training protocols: (1) high-load resistance training (HL-RT); (2) high-load resistance training with blood flow restriction (HL-BFR); and (3) low-load resistance training with blood flow restriction (HL-BFR). Importantly, traditional LL-BFR studies have maintained blood flow restricted (i.e., cuff inflated) throughout training sessions. However, maintaining BFR during an exercise session is very uncomfortable and painful (Fitschen et al., 2014) mainly during HL-BFR. Thus, both BFR protocols (LL-BFR and HL-BFR) maintained blood flow restricted only during the exercise (i.e., cuff was deflated during rest intervals) to maintain the level of blood restriction equalized between protocols (Kacin and Strazar, 2011; Grapar Zargi et al., 2016; Zargi et al., 2018). Pilot worked supported our decision as HL-BFR protocol produced higher local deoxygenation than the HL-RT protocol, while maintaining the total training volume (TTV) equalized between the two groups.

**FIGURE 1:**
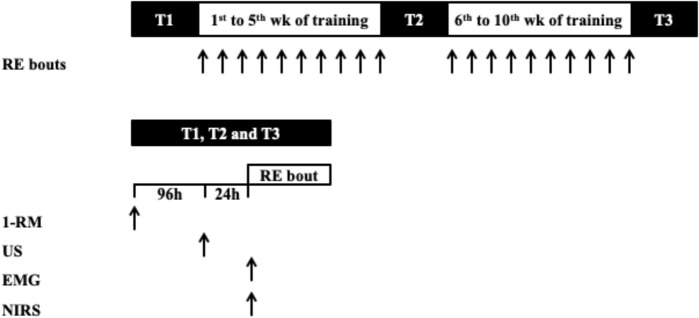
Schematic of study protocol. RE, resistance exercise; 1-RM, one-repetition maximum; EMG, electromyographic; NIRS, near-infrared spectroscopy.

The described dependent variables (i.e., CSA, CSA_echo_/CSA, PA, and 1-RM) were assessed before the experimental protocol (T1), after 5 (T2), and 10 weeks (T3) of the commencement of the experimental period (Figure 1). At T2 and T3 all of the measurements were conducted 72 h after the last training bout performed. Muscle activation and oxygenation were assessed by EMG and near infrared spectroscopy (NIRS), respectively, during a training session in T1, T2, and T3, where each leg performed the training protocol according to the initial randomization.

### Maximum Dynamic Strength Test

Unilateral quadriceps maximum dynamic strength was assessed using the 1-RM test on a leg-extension machine (Effort NKR; Nakagym, São Paulo, Brazil), according to the procedures described elsewhere (Brown and Weir, 2001). In short, participants performed a general warm-up on a cycle ergometer at 20 km⋅h^-1^ for 5 min, followed by specific warm-up sets of leg-extension exercise. In the first set, individuals performed 8 repetitions with a load corresponding to 50% of their estimated 1-RM, obtained during the familiarization sessions. In the second set, they performed three repetitions at 70% of their estimated 1-RM. A 2 min interval was allowed between warm-up sets. After the last warm-up set, a 3 min resting period before the actual 1-RM test. Participants had up to five attempts to achieve their 1-RM load. The smallest increment in load on subsequent attempts was of approximately one kilogram. A 3 min rest interval was allowed between attempts and the highest load achieved (full eccentric–concentric movement with 90° range of motion) was considered as the 1-RM load. The coefficient of variation (CV) and typical error (TE) between two repeated measurements performed 72 h were 1.57% e 1.45 Kg, respectively.

### Determination of the Blood-Flow Restriction Pressure

Before the commencement of the training protocol, blood-flow restriction pressure was determined as follows. Participants in the HL-BFR and LL-BFR protocols were asked to rest comfortably in supine position. A vascular Doppler probe (DV-600; Marted, Ribeirão Preto, São Paulo, Brazil) was placed over the tibial artery to capture its auscultatory pulse. For the determination of blood pressure (mmHg) necessary for complete vascular occlusion (pulse elimination pressure), a standard blood-pressure cuff [175 mm (width) 920 mm (length)] was wrapped around the participant’s thigh at the inguinal fold region and then inflated up to the point at which the auscultatory pulse was interrupted (Libardi et al., 2015a). The pressure in which the auscultatory pulse was interrupted was considered as the occlusion pressure.

### Muscle Cross-Sectional Area

A B-mode ultrasound (US) with a 7.5-MHz linear-array probe (Mysono U6 EX; Samsung-Medison, Gangwon-do, South Korea) was used to capture images in the axial plane of the VL muscle after the participants had laid supine for 20 min to allow for fluid distribution before the assessments (Berg et al., 1993). The image reconstruction technique was used to assess VL muscle CSA following procedures described by our group Lixandrao et al. (2014). In short, the legs were restrained with Velcro^®^ straps to avoid movements of the lower limbs while allowing the participants to relax the leg muscles during the assessments. All the US images were obtained at midpoint between the inferior border of the lateral epicondyle and the greater trochanter of the femur. To increase the accuracy of the measurements at T1, T2, and T3, the skin was marked with semi-permanent ink every 2 cm from the medial to the lateral aspect of the vastus lateralis muscle. Sequential US images were acquired aligning the superior edge of the probe with each of the 2 cm mark, following a middle-to-lateral direction. To avoid deforming the tissue with the pressure applied on the US probe, a generous amount of conductive gel was used. Sequential images of the vastus lateralis muscle were opened in PowerPoint (Microsoft, Redmond, WA, United States), and then each image was manually rotated to reconstruct the whole fascia of the VL muscle. Subsequently, the VL muscle CSA was measured using computerized planimetry, in which the VL muscle CSA was contoured following the muscle fascia using an 800 dpi mouse (Madena 3.2.5; Eye Physics, Los Alamitos, CA, United States). The CV and the TE between two repeated measurements with an interval of 72 h were 0.99% and 0.24 cm^2^, respectively.

### Muscle Cross-Sectional Area Echo Intensity (CSA_echo_)

Muscle CSA previously delimited was analyzed using a Fast Fourier transformation to identify the frequency spectrum of the pixel intensity over the VL CSA (CSA_echo_). This analysis results in a histogram of grayscale shades (0 = black and 256 = white), where any abnormality (e.g., edema-induced muscle swelling, probably due to muscle damage) results in higher echo intensity value (increased white areas), while intact muscle mass presents low echo intensity (i.e., dark areas) (Scanlon et al., 2013; Damas et al., 2016b). Importantly, this method has been extensively used as an indicator of exercise-induced muscle damage edema (Nosaka and Sakamoto, 2001; Chen and Nosaka, 2006; Cheng et al., 2009; Chen et al., 2012, 2013; Gonzalez-Izal et al., 2014; Rosenberg et al., 2014; Damas et al., 2016b). CSA_echo_ was normalized by the vastus lateralis CSA (i.e., CSA_echo_/CSA) to account for the effects of changes in muscle size.

### Pennation Angle (PA)

PA of the VL was measured using the B-mode ultrasound at the thigh mid-point. The PA was defined as the angle between the fascicle and the deep aponeurosis of the VL muscle (Table 1) (Fukunaga et al., 1997). Three consecutive images were obtained and the average value of the PAs was considered for statistical purposes (Blazevich et al., 2007; Alegre et al., 2014; Ando et al., 2014). PA CV and TE between two repeated measurements performed 72 h were 0.035% and 0.670°, respectively.

**Table 1:** Muscle cross section area (CSA), CSA echo intensity (CSAecho), CSAecho to CSA ratio, pennation angle (PA) and maximum dynamic strength test (1-RM) at baseline (T1), after 5 (T2), and 10 weeks (T3) for high-load resistance training (HL-RT), high-load resistance training with blood flow restriction (HL-BFR) and low-load strength training with blood flow restriction (LL-BFR).

Variable	Time	HL-RT	HL-BFR	LL-BFR
CSA (cm^2^)	T1	22.3 ± 6.7	21.8 ± 4.0	21.4 ± 5.8
	T2^∗^	23.4 ± 7.0	22.9 ± 4.3	22.6 ± 6.2
	T3^∗†^	24.5 ± 7.4	24.2 ± 4.7	23.7 ± 6.7
CSA_Echo_ (AU)	T1	19.5 ± 5.1	19.5 ± 5.5	21.0 ± 7.0
	T2^∗^	38.0 ± 9.0	38.2 ± 9.7	40.0 ± 11.0
	T3^†^	17.6 ± 3.3	19. ± 5.5	17.6 ± 4.1
CSA_Echo_ (AU)/CSA (cm^2^)	T1	1.0 ± 0.4	0.9 ± 0.3	1.0 ± 0.4
	T2^∗^	1.7 ± 0.6	1.7 ± 0.4	2.0 ± 0.9
	T3^∗†^	0.7 ± 0.2	0.8 ± 0.3	0.8 ± 0.2
PA (°)	T1	15.0 ± 2.6	15.4 ± 2.0	14.6 ± 2
	T2^∗^	16.0 ± 2.8	16.3 ± 2.0	15.5 ± 2.0
	T3^∗†^	16.4 ± 2.9	17.0 ± 2.1	16.0 ± 2.1
1-RM (kg)	T1	46.6 ± 11.5	46.4 ± 10.8	46.5 ± 11.7
	T2^∗^	55.7 ± 10.8	56.5 ± 10.4	51.3 ± 17.7
	T3^∗†^	63.8 ± 10.9	62.1 ± 18.1	55.2 ± 23.2

*^∗^Significantly different from T1 (main time effect, P < 0.05), ^†^Significantly different from T2 (main time effect, P < 0.05). Values presented as mean ± SD.*

### Knee Joint Angle and Trigger

An angular potentiometer was placed on the right knee of the individuals with its center of rotation aligned with the lateral intercondylar line of the knee joint to determine knee angular excursion and, therefore, the concentric and eccentric phases of the lift. Full extension was defined as “zero degree.” The concentric phase was defined from the maximum to the minimum value of the knee flexion angle, while the eccentric phase was defined from the minimum to the maximum value of the knee flexion angle. The frequency of acquisition was set at 1000 Hz in the A/D converter of the EMG unit described below, which synchronized data acquisition from the angular potentiometer and both the EMG system and the NIRs device. The signal from an external trigger was split and sent to both EMG and NIRs A/D converters to align the data in time.

### Muscle Activation

Muscle activation of the VL muscle, assessed by the amplitude of the electromyographic (EMG) signal (EMG832C; EMG System do Brazil, São José dos Campos, Brazil), was determined at T1, T2, and T3. Before electrode placement, the skin area was shaved, abraded and cleaned with an isopropyl alcohol pad to reduce skin impedance before electrode placement (Libardi et al., 2015b). Pre-gelled Ag/Ag-CL surface electrodes (EMG System, São José dos Campos, Brazil) were placed over the belly of the VL muscle aligned in parallel with the expected muscle fiber orientation and with an interelectrode distance of 2 cm. In addition, a ground electrode was placed in the ankle region at the fibular lateral malleolus. The sampling frequency of the EMG signals was of 1000 Hz with a band-pass filter of 20 and 500 Hz. The EMG amplifiers have an input noise below 1 μV root mean square (RMS) and an effective common rejection mode of 95 dB. Electromyography RMS values were calculated over the concentric phase, defined as the maximum and minimum knee joint angle, on each repetition. Then, RMS values were normalized by the maximal muscle activation (i.e., RMS) obtained during a maximal voluntary isometric contraction (MVIC), calculated over a 250 ms interval around peak torque. After visual inspection, RMS values calculated on each repetition of all training sets (i.e., 1st, 2nd, and 3rd sets) were numerically integrated over time using the trapezoidal rule (GraphPad Prism, GraphPad Software, San Diego, CA, United States), and used for further analysis. The MVIC torque was measured before the exercise protocol having a load cell attached at 90**°** with the lever arm of the leg extension machine. Torque was calculated by the product of the force values by the length of the shank (i.e., distance from the lateral intercondilar line to the lateral malleolus). Load cell data was acquired at a frequency of 1000 Hz using the A/D converter of the EMG unit and digitally filtered with a Butterworth filter set a low pass frequency of 20 Hz. The normalized results of EMG (%MVIC) were presented as mean values of over the concentric phases of the three sets performed during the exercise sessions at T1, T2, and T3.

### Muscle Oxygenation

A continuous dual-wavelength near-infrared spectroscopy apparatus (NIRS; Oxymon, Artinis Medical Systems, Arnhem, the Netherlands) was used to monitor changes in muscle oxygenation during the HL-RT, HL-BFR, and LL-BFR protocols in T1, T2, and T3 assessments. Data was collected at a frequency of 25 Hz. The system uses a modified Beer-Lambert law to analyze the changes in light absorbed at wave lengths of 761 and 844 nm, estimating concentrations of deoxygenated hemoglobin ([HHb]), which has been considered an indicator of metabolic stress (Cayot et al., 2016; Lauver et al., 2017).

Initially, thickness of the subcutaneous fat layer at the site of NIRs optodes (i.e., an emitter and a detector) placement was assessed by ultrasound (HL: 0.83 ± 0.18 cm, HL-BFR: 0.91 ± 0.22 cm, and LL-BFR: 0.87 ± 0.20 cm) to set the value of laser penetration depth (Koga et al., 2007). Following, a large area of skin was shaved and cleaned with alcohol. Then, the holder of the pair of optodes was fixed on the skin with adhesive tapes. The optodes were positioned in the VL muscle, 3 cm medial from the point used to fix EMG electrodes (Ferrari et al., 2004). Before each data collection, the equipment was set to a sampling frequency of 25 Hz (Kacin and Strazar, 2011).

Data were extracted from the NIRS device using Oxisoft (3.0.X; Artinis Medical Systems B.V, Arhem, Netherlands) and a customized script analyzed the data off-line. Raw data was filtered with a moving average algorithm over a 2 s period. Then, [HHb] resting values were obtained during the last 5 min of a 15 min rest period, in which individuals remained still and as relaxed as possible prior to exercise commencement (Tanimoto and Ishii, 2006; Kon et al., 2010; Callewaert et al., 2013). Exercise values were determined as the difference between the [HHb] values, obtained over the concentric phase of each repetition (as described above), and the mean [HHb] value over the 5 min resting period. [HHb] values calculated on each repetition of all training sets (i.e., 1st, 2nd, and 3rd sets) were numerically integrated over time using the trapezoidal rule (GraphPad Prism, GraphPad Software, San Diego, CA, United States), for each of the assessments (i.e., T1, T2, and T3) (Ganesan et al., 2015), and used for further analysis.

### Resistance Training Protocols

Training protocols were performed unilaterally using a conventional leg-extension machine, twice a week for 10 weeks. The HL-RT and HL-BFR protocols performed 3 sets of 10 repetitions with a load corresponding to 80% 1-RM (HL-RT: T1–T2 = 37.2 ± 9.2 kg and T2–T3 = 44.5 ± 8.6 kg; HL-BFR: T1–T2 = 37.1 ± 8.7 kg and T2–T3 = 45.2 ± 8.4 kg), while the LL-BFR protocol performed 3 sets of 20 repetitions with 20% 1-RM (LL-BFR: T1–T2 = 9.3 ± 2.3 kg and T2–T3 = 10.2 ± 3.5 kg). A 1 min rest period was granted between sets for all of the protocols. After the fifth week (10th session), 1-RM was re-assessed to adjust training load. From week 6 (T2–T3) onwards, the number of sets was increased to four, for all of the participants. The cuff pressure used during the BFR protocols was set at 60% of occlusion pressure in the resting condition. The cuff pressure remained inflated during the exercise and deflated during the rest periods. The average pressure used throughout the training protocol was 81.85 ± 4.45 mmHg.

### Statistical Analysis

After visual inspection, the area under the curve (AUC) analysis for EMG and [HHb] were performed using the trapezium rule (GraphPad Prism, GraphPad Software, San Diego, CA, United States) in order to characterize the magnitude of the response and the changes over time. AUC analyses were calculated using the time point immediately before (Pre) and changes in EMG and [HHb] in the 1st, 2nd, and 3rd sets. The 1-RM, CSA, CSAecho, CSAecho/CSA, PA, EMG, and [HHb] data were analyzed using mixed models having training protocol and time as fixed factors, and subjects as random factor. Only TTV was analyzed with a one-way repeated measures model having training protocol as a fixed factor and subjects as a random factor. In case of significant values of *F*, a Fisher’s LSD *post hoc* analysis was used for multi comparison purposes. Pearson correlation was used to estimate the association between changes in [HHb] (Average of the values of T1 and T2, T2 and T3, T1 and T3 multiplied by the number of sessions in the same periods) and muscle CSA (Percentage change from T1 to T2, T2 to T3, and T1 to T3). All statistical analyses were performed using SAS software (SAS Institute Inc, Cary, NC, United States). Effect sizes (ES) were calculated for 1-RM and muscle CSA using the changes from T1 to T3. ES were classified as “small” if lower than 0.2, “medium” if between 0.2 and 0.5, and “large” if higher than 0.8 (Cohen, 1988). Significance level was set at *P* < 0.05 and data was presented as mean and standard deviation (SD).

## Results

### Total Training Volume (TTV)

TTV (sets × repetitions × load [kg]) in the LL-BFR (11733.0 ± 3204.9 kg) was lower than HL-RT (24546.0 ± 5329.0 kg, *P* < 0.0001) and HL-BFR (24708.0 ± 4992.4 kg; *P* < 0.0001) protocols. No significant differences in TTV were detected between the HL-RT and HL-BFR protocols (*P >* 0.05).

### Maximum Dynamic Strength Test (1-RM)

1-RM values increased, similarly, and significantly for the HL-RT, HL-BFR, and LL-BFR groups from T1 to T2 (main time effect *P* < 0.0001) and from T1 to T3 [ES: HL-RT, 1.24 (large); HL-BFR, 1.42 (large) and LL-BFR, 0.96 (large); main time effect, *P* < 0.0001]. In addition, 1-RM values at T3 were significantly greater than that at T2 (main time effect *P* < 0.0001) (Table 1).

### Muscle Cross-Sectional Area (CSA)

The HL-RT, HL-BFR, and LL-BFR protocols groups showed significant and similar increases in muscle CSA from T1 to T2 (main time effect, *P* < 0.0001) and from T1 to T3 [ES: HL-RT, 0.26 (moderate); HL-BFR, 0.46 (moderate), and LL-BFR, 0.30 (moderate); main time effect, *P* < 0.0001]. There was also a significant increase from T2 to T3 (main time effect, *P* < 0.0001) (Table 1).

### CSA Echo Intensity (CSA_echo_)

CSA_echo_ analysis (Table 1) showed significant increases from T1 to T2 (main time effect, *P* < 0.0001), and significant decreases from T2 to T3 (main time effect, *P* < 0.0001). There were no significant changes in CSA_echo_ from T1 to T3 (*P* > 0.05). When CSA_echo_ was normalized by VL CSA (CSA_Echo_/CSA), T2 was significantly elevated in all three conditions compared to T1 (main time effect, *P* < 0.0001) and T3 (main time effect *P* < 0.0001). In addition, T3 showed significantly lower values than T1 for the three protocols (main time effect *P* < 0.0001).

### Pennation Angle (PA)

In relation to the PA (Table 1), HL-RT, HL-BFR, and LL-BFR protocols produced significant increases from T1 to T2 (main time effect, *P* < 0.0001) and from T1 to T3 (main time effect *P* < 0.0001). There were also significant increases from T2 to T3 for all groups (main time effect, *P* < 0.0001).

### Muscle Activation

**FIGURE 2:**
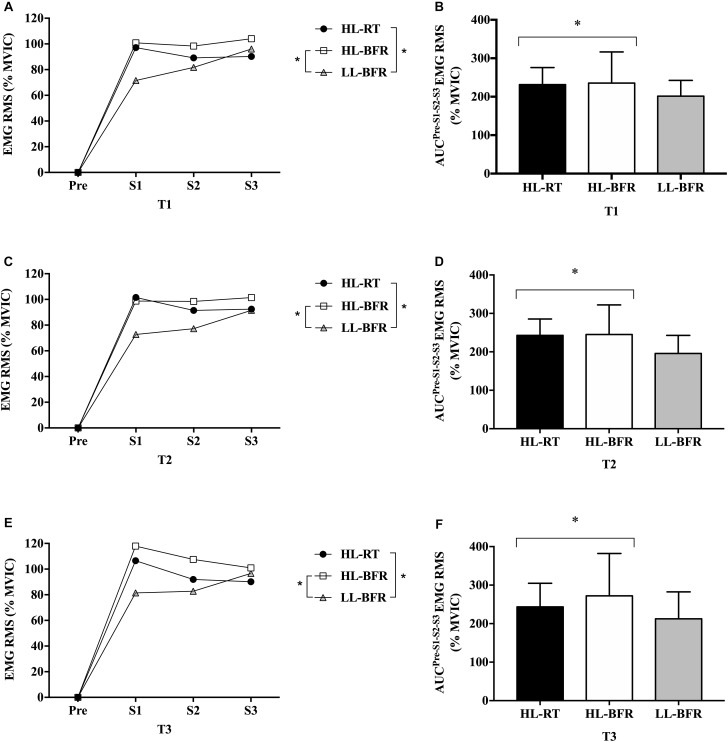
Normalized electromyographic (EMG) root mean square (RMS) values from the resistance training session (Pre [Before the beginning of the exercise], set 1 [S1], set 2 [S2], and set 3 [S3]) at baseline (T1, **A**), after 5 (T2, **C**) and 10 weeks (T3, **E**) for the high-load resistance training (HL-RT), high-load resistance training with blood flow restriction (HL-BFR) and low-load resistance training with blood flow restriction (LL-BFR) protocols. EMG RMS values also are reported as area under the curve (AUC) from the entire resistance training session at T1 **(B)**, T2 **(D)**, and T3 **(F)** for the HL-RT, HL-BFR and LL-BFR protocols. ^∗^Significantly different from LL-BFR (main protocol effect, *P* < 0.01). Values presented as mean ± SD.

EMG amplitude AUC was greater during HL-RT and HL-BFR than LL-BFR (main protocol effect, *P* = 0.01 and *P* = 0.001), with no differences between HL-RT and HL-BFR (*P >* 0.05) (Figure 2). Additionally, EMG amplitude AUC was not different between T1, T2, and T3 (*P >* 0.05) (Figure 2).

### Muscle Oxygenation

**FIGURE 3:**
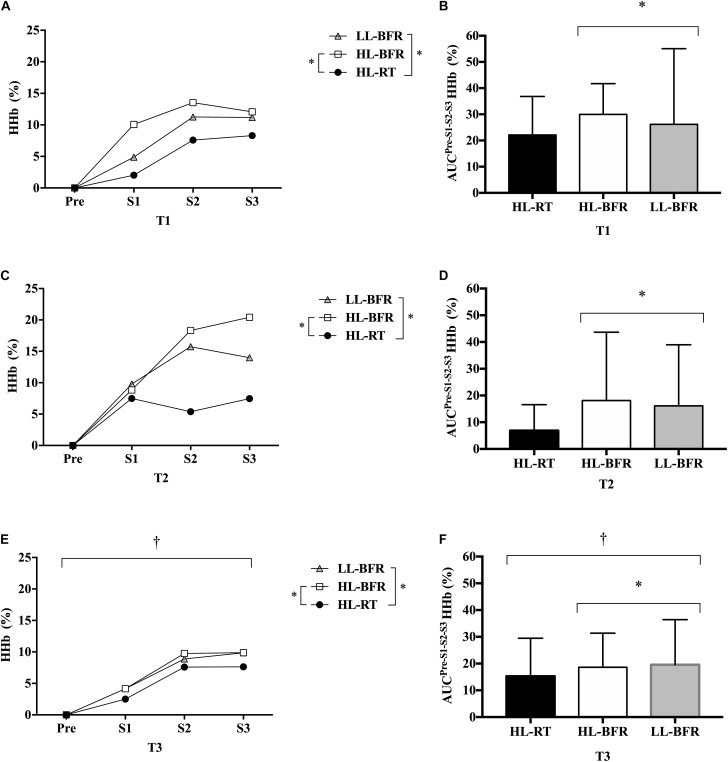
Normalized deoxygenated hemoglobin ([HHb]) values from the resistance training session (Pre [Before the beginning of the exercise], set 1 [S1], set 2 [S2], and set 3 [S3]) at baseline (T1, **A**), after 5 (T2, **C**) and 10 weeks (T3, **E**) for the high-load resistance training (HL-RT), high-load resistance training with blood flow restriction (HL-BFR) and low-load resistance training with blood flow restriction (LL-BFR) protocols. [HHb] values also are reported as area under the curves (AUC) from the entire resistance training session at T1 **(B)**, T2 **(D)**, and T3 **(F)** for the HL-RT, HL-BFR, and LL-BFR protocols. ^∗^Significantly different from HL-RT (main protocol effect, *P* < 0.04). ^†^Significant different from T2 (main time effect, *P* < 0.04). Values presented as mean ± SD.

The [HHb] AUC was significantly lower for all protocols in T3 compared to T2 (main time effect, *P* = 0.04), but similar to T1. No differences were observed between T1 and T2 (*P >* 0.05). Regarding protocol comparison, [HHb] AUC was greater during HL-BFR and LL-BFR than HL-RT (main protocol effect, *P* = 0.02 and *P* = 0.03), with no difference between HL-BFR and LL-BFR (*P >* 0.05) (Figure 3).

There was a significant correlation between [HHb] and CSA area only at T2–T3 for the LL-BFR protocol (*r* = 0.71; *P* = 0.0008) (Table 2). No significant correlations were found between [HHb] and CSA for the HL-RT and HL-BFR protocols (*P* > 0.05). Additionally, we collapsed the groups to test overall significant correlations between muscle oxygenation variables and hypertrophy response and no significant correlations were observed (*P* > 0.05).

**Table 2:** Correlations between changes in deoxyhemoglobin concentrations ([HHb]) and changes in muscle cross-sectional area (CSA) for high-load resistance training (HL-RT), high-load resistance training with blood flow restriction (HL-BFR), low-load blood flow restriction (LL-BFR) and all groups together.

Variable		CSA (%) (T1–T2)	CSA (%) (T2–T3)	CSA (%) (T1–T3)
HL-RT [HHb] (T1–T2)	*r*	0.30		
	*P*	0.19		
HL-RT [HHb] (T2–T3)	*r*		0.14	
	*P*		0.56	
HL-RT [HHb] (T1–T3)	*r*			0.03
	*P*			0.90
HL-BFR [HHb] (T1–T2)	*r*	0.136		
	*P*	0.580		
HL-BFR [HHb] (T2–T3)	*r*		0.43	
	*P*		0.08	
HL-BFR [HHb] (T1–T3)	*r*			0.05
	*P*			0.86
LL-BFR [HHb] (T1–T2)	*r*	0.10		
	*P*	0.67		
LL-BFR [HHb] (T2–T3)	*r*		0.71	
	*P*		0.0008^∗^	
LL-BFR [HHb] (T1–T3)	*r*			0.36
	*P*			0.22
All protocols [HHb] (T1-T2)	*r*	0.05		
	*P*	0.71		
All protocols [HHb] (T2–T3)	*r*		0.09	
	*P*		0.23	
All protocols [HHb] (T1–T3)	*r*			0.05
	*P*			0.75

*^∗^Significant correlation (P < 0.05).*

## Discussion

We aimed to compare the effects of HL-RT, HL-BFR, and LL-BFR on muscle deoxygenation (HHb), CSA, activation, strength, architecture, and edema before (T1), after 5 (T2), and 10 weeks (T3) of training. Additionally, we analyzed the association between [HHb] and muscle hypertrophy. Regarding our four hypotheses, we confirmed that: (1) BFR did not produce an additive effect on muscle hypertrophy when mechanical tension is high; (2) high mechanical tension protocols did not produce a significant correlation between metabolic stress and muscle hypertrophy (i.e., HL-RT and HL-BFR); (3) LL-BFR produced similar neuromuscular adaptations to HL-RT; and (4) metabolic stress has a positive and significant association with muscle hypertrophy only when mechanical tension is low (i.e., LL-BFR). Thus, mechanical tension and metabolic stress seem to share the variance of the muscle hypertrophy response under high mechanical tension protocols, while metabolic stress seems to be the main mechanism responsible for muscle hypertrophy when mechanical tension is low.

### Muscle Strength

Regarding muscle strength, previous studies have reported similar increases in muscle strength between HL-RT and LL-BFR protocols (Takarada et al., 2000; Karabulut et al., 2010; Laurentino et al., 2012). For instance, Laurentino et al. (2012) demonstrated significant and similar increases in 1-RM values after 12 weeks in HL-RT and LL-BFR protocols (36.2 and 40.1%, respectively). In our study, muscle strength increased significantly after 10 weeks of training (HL-RT = 41.0% and LL-BFR = 32.2%, respectively). Thus, the increases in muscle strength after LL-BFR protocol are comparable to previous studies (Laurentino et al., 2012). Increments in muscle strength in LL-BFR protocols are usually attributed to the maintenance of BFR throughout the training session (exercise and pause between sets). The findings reported herein show otherwise, as we deflated the BFR cuff during the resting intervals. Furthermore, our findings support previous ones (Laurentino et al., 2008) as BFR did not produce additive effects on strength gains when mechanical tension was high (i.e., HL-BFR). Taken together, these findings are of great importance for the viability of LL-BFR protocols, as when BFR is applied only during exercise, it promotes lower perception of pain compared to traditional BFR protocols (Fitschen et al., 2014).

### Muscle Hypertrophy

Usually, studies have reported that low-load RT promotes a small or even no increase in the muscle CSA (Takarada et al., 2000; Yasuda et al., 2010; Laurentino et al., 2012). However, our group and others (Takarada et al., 2002; Laurentino et al., 2012; Yasuda et al., 2012, 2013; Vechin et al., 2014; Libardi et al., 2015a; Lixandrao et al., 2015) have shown that the addition of BFR to low-load RT produces increases in muscle CSA (between 6 and 7.5%), comparable to HL-RT (between 6 and8%), after a 12 weeks training. In our study, we found significant and similar increases in CSA after 5 and 10 weeks of HL-RT (4.9 and 10.0%, respectively) and LL-BFR (5.0 and 10.0%, respectively). Despite the release of BFR cuff pressure during the rest interval between sets, our results suggest that muscle hypertrophy response is not affected when compared to LL-BFR protocols in which BFR cuff is maintained inflated throughout the training session. Regarding the comparison between HL-RT and HL-BFR, our results demonstrated that the addition of BFR does not result in additional increases in muscle CSA, as these protocols presented virtually the same changes in VL CSA at T2 (4.98 and 5.19%, respectively) and T3 (10.01 and 10.36%, respectively) compared to T1. Although only one study has investigated the effects of HL-BFR, Laurentino et al. (2008) reported similar increases in quadriceps CSA for HL-RT and HL-BFR after 8 weeks of training (6.1 and 5.0%, respectively).

Importantly, muscle hypertrophy was accompanied by increases in pennation angle (PA). These increases occurred in the early stages of training [T2, 5 weeks (10 training sessions)] and were even greater at T3 [10 weeks (20 training sessions)], as shown in other studies (Seynnes et al., 2007). However, in the present study, the increases in CSA and PA were accompanied by higher CSA_echo_ in T2 than T1, followed by a reduction in T3. These results indicate that muscle hypertrophy and changes in muscle architecture observed in T2 were likely related to edema/muscle damage, and not to the expansion of myofibrillar content. Recently, our group demonstrated that after 2 weeks of RT, increases in muscle CSA were accompanied by enhanced CSA_echo_ and systemic markers of muscle damage (e.g., myoglobin and interleukin-6) (Damas et al., 2016b). Accordingly, it has been shown that only a single HL-RT or LL-BFR bout can change the muscle architecture due to edema caused by exercise-induced muscle damage (Kubo et al., 2006; Martin-Hernandez et al., 2013; Yu et al., 2015). To support this idea, we normalized the CSA_echo_ by CSA (i.e., CSA_Echo_/CSA) and the results demonstrated that T2 values were also significantly higher than T1 and T3 ones. Supporting our previous findings (Damas et al., 2016b), we observed a significant decrease in CSA_echo_ with concomitant increases in CSA and PA at T3. These results suggest that after 20 training sessions (i.e., 10 weeks), muscle damage-related changes in muscle morphology are attenuated, and muscle hypertrophy produced the changes observed in morphology. Furthermore, the strong association between changes in intramuscular [Pi] (i.e., metabolic stress marker) and muscle CSA (*r* = 0.87), reported by Takada et al. (2012) may have been induced by muscle edema, rather than actual muscle hypertrophy, as it artificially increases muscle CSA (Damas et al., 2016b).

### Muscle Activation

It has been suggested that muscle strength gains and hypertrophy after a RT period, with or without BFR, are associated with increases in the ability to activate the motor unit pool (Suga et al., 2012; Schoenfeld, 2013) as assessed by the amplitude of the surface EMG signal (Takarada et al., 2000; Laurentino et al., 2008; Sundstrup et al., 2012). However, few studies have investigated the changes in EMG amplitude during the actual training protocol (Kacin and Strazar, 2011). In the present study, we observed higher EMG amplitude in T1, T2, and T3 for HL-RT and HL-BFR compared to LL-BFR. Considering the comparison between HL-RT and LL-BFR, some acute studies (i.e., a single training session) also showed that EMG values were higher for HL-RT compared to LL-BFR (Kubo et al., 2006; Manini and Clark, 2009). Regarding HL-RT and HL-BFR, to our knowledge, only one study compared the EMG amplitude between HL-RT and HL-BFR, and found no significant differences between these protocols after a single bout (Neto et al., 2014). Collectively, these results suggest that the recruitment of MUs does not seem to explain the similarity in muscle strength and hypertrophy gains between these protocols. Limitations of the EMG assessment (Dimitrova and Dimitrov, 2003) and other possible mechanisms may have contributed to these findings.

### Muscle Oxygenation

It has been suggested that [HHb] is a proxy marker of metabolic stress (Cayot et al., 2016; Lauver et al., 2017), which has been considered as a primary stimulus to muscle growth (Schoenfeld, 2010, 2013; Pearson and Hussain, 2015). Accordingly, studies have used BFR during low-load RT to increase metabolic stress, and to induce muscle hypertrophy response (Takada et al., 2012). However, little is known regarding the role of metabolic stress in high mechanical tension (e.g., HL-RT) protocols and if increases in metabolic stress may produce and additive effect on muscle hypertrophy response. In this study, we added BFR to both low- and high-load RT and compared them with HL-RT, traditionally recommended to maximize increases in muscle strength and hypertrophy (American College of Sports Medicine [ACSM], 2009, 2011). Metabolic stress can be measured by near-infrared spectroscopy (NIRS) during exercise, as it non-invasively identifies changes in the relative concentrations of deoxyhemoglobin ([HHb]) in muscle tissue. Regarding, the comparison between time points, we observed that after 10 weeks (T3) of training, increases in [HHb] were lower than at T2 (5 weeks) and similar to T1 for all protocols. In this sense, Kacin and Strazar (2011) investigated the effects of 4 weeks of low-load RT with and without BFR during knee-extension exercise at 15% maximal voluntary muscle contraction to the voluntary failure. The results show attenuated decrease in concentrations of oxygenated hemoglobin ([HbO_2_]) after the training period for both experimental conditions. Although studies have observed changes in different markers throughout the RT, both suggest a peripheral adaptation probably induced by local angiogenesis responses to RT protocols. Indeed, RT protocols might increase vascular endothelial growth factor expression and other transcription factors/growth associated with the formation of capillaries (i.e., angiogenesis) (Gavin et al., 2007).

Comparing the protocols, those performed with BFR (i.e., HL-BFR and LL-BFR) showed higher deoxygenation compared to HL-RT. Interestingly, notwithstanding HL-BFR combine the highest levels of metabolic stress and mechanical tension, the muscle hypertrophy and strength gains were similar to protocols with lower levels of metabolic stress (i.e., HL-RT) or mechanical tension (i.e., LL-BFR). These results may suggest that muscle protein synthesis may reach maximal values when training with high intensities (e.g., 80% 1-RM – high mechanical tension) and the metabolic stress does not seem to produce additive effects to muscle hypertrophy. On the other hand, in low-load protocols (e.g., 30% 1-RM) the metabolic stress induced by BFR seems to fully activate the muscle protein synthesis machinery. This is supported by the strong association between changes in [HHb] and changes in muscle CSA only to LL-BFR group (*P* = 0.008; *r* = 0.716). Further studies are required to elucidate this issue.

### Limitations

(1) We show greater muscle deoxygenation for HL-BFR and LL-BFR and higher muscle activation for HL-RT and HL-BFR, but with similar neuromuscular adaptations between these protocols in untrained men. Although one may rightly suggest that these findings may not be directly extended to trained individuals, to the best of our knowledge, there is no empirical evidence suggesting that the activation of specific hypertrophy triggers may change as a function of the training status.
(2) A high and significant correlation between [HHb] and muscle hypertrophy were showed for LL-BFR. However, these findings should be viewed with caution, as other NIRS parameters, such as deoxyhemoglobin (HbO_2_), total hemoglobin (HBT) and hemoglobin difference (Hbdiff) were not analyzed in the present study. In addition, the agreement between [HHb] and other parameters related to metabolic stress (e.g., phosphocreatine, inorganic phosphate, muscle pH, and lactate) should be analyzed in future studies to confirm our findings. On the other hand, the HHb is highly correlated with blood lactate (Grassi et al., 2003), which has been traditionally considered as a proxy marker of metabolic stress (Cayot et al., 2016; Lauver et al., 2017). Furthermore, it has been previously reported that the [HHb] signal is less sensitive to changes in blood volume compared to other markers related to muscle oxygenation (Ferrari et al., 1997). Thus, [HHb] seems to be a better index to compare protocols with BFR, as they may produce large fluctuations in blood volume.
(3) We also suggest that future studies should investigate the effects of secondary hypertrophy-related mechanisms triggered by metabolic stress (e.g., increased fast-twitch fiber recruitment, local hormone, cell swelling, and the production of reactive oxygen species) on muscle hypertrophy to further elucidate these complex mechanisms.
(4) It is also important to consider that the findings reported herein should be confirmed in other BFR training protocols, as it has been demonstrated that metabolic stress may change as a function of the occlusion pressure and training load (Suga et al., 2010).
(5) BFR training pressure was determined in a resting state, which may be considered as an inherited limitation of the method, as pressure fluctuates during the eccentric and concentric phases of the lifts.

### Physiological Relevance

Muscle deoxygenation seems to play an important role on neuromuscular adaptations when RT is performed with low-load, as it produces similar neuromuscular adaptions to high-load protocols, despite the lower (TTV) (∼53%). Corroborating this suggestion, there was a significant association between the changes in [HHb] and increases in muscle CSA from T2 to T3, when muscle edema was attenuated. We propose that the level of metabolic stress would not influence the magnitude of muscle hypertrophy, as well as changes in muscle strength and architecture, when RT is performed in high-loads.

## Conclusion

Our results suggest that the addition of BFR to exercise contributes to neuromuscular adaptations only when RT is performed with low-load. Furthermore, we found a significant association between the changes in [HHb] (i.e., metabolic stress) and increases in muscle CSA from T2 to T3 only for the LL-BFR, when muscle edema was attenuated.

## Ethics Statement

This study was carried out in accordance with the recommendations of ethics committee of Federal University of São Carlos (UFSCar), number 42359015.5.0000.5504 with written informed consent from all subjects. All subjects gave written informed consent in accordance with the Declaration of Helsinki. The protocol was approved by the ethics committee of Federal University of São Carlos (UFSCar).

## Author Contributions

CAL had the original idea of the study and the final study design was developed by CAL, CU, TMPCB, and AB-S. Participants were recruited, trained and assessed at the Federal University of São Carlos, by TMPCB, RMO, SDS, and JGB. TMPCB, CAL, and CU performed data analyses and statistical procedures and wrote the first version of the manuscript. All authors participated in the interpretation of the data, contributed to the revision of the manuscript, and approved the content of the final version.

## Conflict of Interest Statement

The authors declare that the research was conducted in the absence of any commercial or financial relationships that could be construed as a potential conflict of interest.
